# O Papel da Impressão 3D na Cirurgia Cardíaca para Doenças Cardíacas Congênitas

**DOI:** 10.36660/abc.20240798

**Published:** 2025-01-30

**Authors:** Vitor Ramos

**Affiliations:** 1 Leeds Teaching Hospitals NHS Trust Leeds General Infirmary Leeds Reino Unido Leeds Teaching Hospitals NHS Trust – Paediatric Cardiology, Leeds General Infirmary, Leeds – Reino Unido

**Keywords:** Cardiopatias Congênitas, Impressão Tridimensional, Cirurgia Torácica

A impressão 3D está revolucionando várias indústrias, permitindo a criação de modelos físicos intrincados e personalizados por meio de camadas de materiais como silicone ou polímeros com base em designs digitais. Entre suas diversas aplicações, a área da saúde tem visto avanços significativos com essa tecnologia.^1^

Em cardiologia, a adoção da modelagem 3D ficou para trás em comparação a outros campos, principalmente devido ao desafio de retratar a natureza complexa e dinâmica do coração. Esse padrão reflete a lenta integração inicial da tomografia computadorizada e da ressonância magnética no tratamento cardíaco — tecnologias que foram amplamente utilizadas em especialidades não cardíacas antes de transformar o diagnóstico e o tratamento cardiovascular.^2,3^ Na última década, no entanto, a impressão 3D surgiu como uma ferramenta transformadora na cirurgia cardíaca, aprimorando a visualização, o planejamento pré-operatório e os resultados dos pacientes.^1^

Ao criar modelos físicos precisos do coração de um paciente, a impressão 3D permite que os cirurgiões obtenham uma compreensão precisa das estruturas cardíacas individuais, o que a torna especialmente valiosa para o tratamento de doenças cardíacas congênitas (DCC).^4^

O tratamento da DCC tem visto um rápido progresso, com avanços em técnicas cirúrgicas e baseadas em cateteres melhorando drasticamente as taxas de sobrevivência. Mais de 95% das crianças nascidas com DCC agora sobrevivem até a idade adulta. No entanto, apesar desses avanços, uma cura completa continua rara. Em vez disso, a morbidade e a mortalidade estão cada vez mais sendo transferidas para a idade adulta, pois os pacientes com DCC geralmente requerem cuidados ao longo da vida.^5^

Um desafio significativo nas intervenções de DCC é a prevalência de cirurgias paliativas anteriores, muitas das quais se tornaram obsoletas por técnicas modernas. Os cirurgiões devem, portanto, permanecer proficientes não apenas nos últimos avanços, mas também em procedimentos históricos para navegar neste campo complexo. Pacientes com DCC formam um grupo altamente heterogêneo com ampla variabilidade dentro de pequenas populações de pacientes. Consequentemente, o gerenciamento individualizado é crítico, contando com representações diagnósticas precisas, particularmente para intervenções complexas.^6^

Embora a imagem transversal e a ecocardiografia avançada sejam indispensáveis no tratamento de DCC, elas frequentemente não conseguem transmitir os detalhes intrincados necessários para cirurgias complexas ou procedimentos baseados em cateter.^7^ A utilidade desses métodos de imagem depende muito de quão bem os dados processados, tanto análises quantitativas quanto representações anatômicas visuais, são comunicados a especialistas não especialistas em imagem. É aqui que a impressão 3D se destaca como uma ferramenta translacional, preenchendo lacunas de entendimento entre equipes multidisciplinares e facilitando a colaboração entre especialistas em imagem, cirurgiões e cardiologistas intervencionistas.^8^

Curiosamente, o impacto dos modelos 3D parece menos pronunciado para cirurgiões experientes do que para cirurgiões juniores, ajudando os últimos a construírem confiança e experiência nesta subespecialidade desafiadora.^9^ Alguns centros já estão usando modelos impressos em 3D para treinamento cirúrgico, o que é particularmente importante, pois muitas instituições enfrentam dificuldades para substituir cirurgiões aposentados com experiência comparável.

Além das aplicações cirúrgicas, os modelos impressos em 3D podem servir como ferramentas de comunicação poderosas, ajudando pacientes e familiares a entenderem melhor condições complexas. Isso promove confiança e auxilia na tomada de decisões informadas, embora as evidências desses benefícios permaneçam um tanto inconsistentes.^10^

Apesar de suas vantagens teóricas, as evidências que apoiam a impressão 3D no tratamento cardíaco são tristemente limitadas, especialmente no campo da DCC, onde faltam revisões sistemáticas. A raridade e a heterogeneidade da DCC, que a tornam uma candidata ideal para impressão 3D, paradoxalmente complicam os esforços para reunir dados robustos sobre sua eficácia. É bem-vindo que os autores do estudo^11^ tenham assumido o desafio de conduzir uma revisão completa dos benefícios da tecnologia de impressão 3D, conduzindo uma metanálise de 21 estudos (444 pacientes no total) publicados até o início deste ano.^11^ Eles descobriram que a impressão 3D alterou os planos cirúrgicos iniciais em cerca de metade dos casos (51,8% dos casos, IC de 95% 26,6–77,0%, p=0,001), o que é notável e de impacto obviamente importante no tratamento do paciente. Embora tenham sido observadas reduções no tempo operatório, na duração da ventilação mecânica e nas internações na UTI, essas descobertas não foram estatisticamente significativas. No entanto, os autores do estudo^11^ destacaram os vieses da análise, incluindo os pequenos tamanhos de estudo e o viés de seleção, já que a impressão 3D é normalmente usada para um pequeno subconjunto de pacientes com DCC. Restrições de tempo e custo limitam ainda mais a adoção generalizada dessa tecnologia, mesmo em departamentos com bons recursos. Como os autores do estudo^11^ concluíram corretamente, suas descobertas precisam ser confirmadas em estudos e metanálises adicionais com números maiores de casos, e estudos randomizados para a aplicação da tecnologia são necessários para reunir evidências robustas.

Tecnologias emergentes em realidade virtual e aumentada (RV/RA) podem oferecer uma alternativa econômica e eficiente à impressão 3D tradicional de modelos físicos. Essas aplicações transformam dados DICOM brutos de modalidades de imagem em modelos virtuais, permitindo intervenções simuladas, como projetar remendos cirúrgicos ou stents. Ao reduzir o tempo e as despesas associadas à criação de modelos físicos, a RV/RA tem o potencial de expandir o acesso à modelagem 3D em mais departamentos médicos e pacientes em todo o mundo.^12,13^
