# Supplementary material for: O Papel da Impressão 3D na Cirurgia Cardíaca para Doenças Cardíacas Congênitas
Source: Arq Bras Cardiol. 2025 Jan 30;121(12):e20240798. [Article in Portuguese] doi: 10.36660/abc.20240798 (PMC11809804; doi:10.36660/abc.20240798)
Supplement: Translation

# Translation: The Role of 3D Printing in Cardiac Surgery for Congenital Heart Diseases

**DOI:** 10.36660/abc.20240798i

**Authors:** Vitor Ramos

**Affiliations:** 1 Leeds United Kingdom Leeds Teaching Hospitals NHS Trust – Paediatric Cardiology, Leeds General Infirmary, Leeds – United Kingdom

**Keywords:** Congenital Heart Defects, Three-Dimensional Printing, Thoracic Surgery

3D printing is revolutionizing various industries, enabling the creation of intricate, customized physical models by layering materials such as silicone or polymers based on digital designs. Among its diverse applications, healthcare has seen significant advancements with this technology.^1^

In cardiology, the adoption of 3D modeling has lagged compared to other fields, primarily due to the challenge of depicting the heart's complex, dynamic nature. This pattern mirrors the initial slow integration of computed tomography and magnetic resonance imaging into cardiac care — technologies that were first widely used in non-cardiac specialties before transforming cardiovascular diagnosis and management.^2,3^ Over the last decade, however, 3D printing has emerged as a transformative tool in cardiac surgery, enhancing visualization, preoperative planning, and patient outcomes.^1^

By creating accurate physical models of a patient's heart, 3D printing allows surgeons to gain a precise understanding of individual cardiac structures, making it especially valuable for managing congenital heart disease (CHD).^4^

The management of CHD has seen rapid progress, with advancements in surgical and catheter-based techniques dramatically improving survival rates. Over 95% of children born with CHD now survive into adulthood. However, despite these strides, a complete cure remains rare. Instead, morbidity and mortality are increasingly shifted to adulthood, as CHD patients often require lifelong care.^5^

One significant challenge in CHD interventions is the prevalence of prior palliative surgeries, many of which have been rendered obsolete by modern techniques. Surgeons must, therefore, stay proficient not only in the latest advances but also in historical procedures to navigate this complex field. CHD patients form a highly heterogeneous group with wide variability within small patient populations. Consequently, individualized management is critical, relying on precise diagnostic representations, particularly for complex interventions.^6^

While cross-sectional imaging and advanced echocardiography are indispensable in CHD care, they often fall short in conveying the intricate details required for complex surgeries or catheter-based procedures.^7^ The utility of these imaging methods heavily depends on how well the processed data, both quantitative analyses and visual anatomical representations, are communicated to non-imaging specialists. This is where 3D printing excels as a translational tool, bridging gaps in understanding among multidisciplinary teams and facilitating collaboration between imaging specialists, surgeons, and interventional cardiologists.^8^

Interestingly, the impact of 3D models seems less pronounced for experienced surgeons than for junior surgeons, helping the latter to build confidence and expertise in this challenging subspecialty.^9^ Some centers are already using 3D-printed models for surgical training, which is particularly important as many institutions face difficulties replacing retiring surgeons with comparable expertise.

Beyond surgical applications, 3D-printed models can serve as powerful communication tools, helping patients and families better understand complex conditions. These fosters trust and aid informed decision-making, though evidence for these benefits remains somewhat inconsistent.^10^

Despite its theoretical advantages, the evidence supporting 3D printing in cardiac care is sadly limited, especially in the CHD field, where systematic reviews are lacking. The rarity and heterogeneity of CHD, which make it an ideal candidate for 3D printing, paradoxically complicate efforts to gather robust data on its efficacy. It is welcomed that the study^11^ took the challenge to conduct a thorough review of the benefits of 3D printing technology by conducting a meta-analysis of 21 studies (444 patients in total) published up to the start of this year.^11^ They found that 3D printing altered initial surgical plans in around half of the cases (51.8% of cases, 95% CI 26.6–77.0%, p=0.001), which is remarkable and of obvious major impact on the patient's care. While reductions in operative time, mechanical ventilation duration, and ICU stays were observed, these findings were not statistically significant. Nevertheless, the study^11^ highlighted the analysis biases, including the small study sizes and selection bias, as 3D printing is typically used for a smallish subset of CHD patients. Time and cost constraints further limit the widespread adoption of this technology, even in well-resourced departments. As the study^11^ authors correctly conclude, their findings need to be confirmed in further studies and meta-analyses with larger numbers of cases, and randomized studies for the application of technology are needed to gather robust evidence.

Emerging technologies in virtual and augmented reality (VR/AR) may offer a cost-effective and efficient alternative to traditional 3D printing of physical models. These applications transform raw DICOM data from imaging modalities into virtual models, enabling simulated interventions such as designing surgical patches or stents. By reducing the time and expense associated with creating physical models, VR/AR has the potential to expand access to 3D modeling across more medical departments and patients worldwide.^12,13^
